# Severe allergic rash induced by icodextrin: case report and literature review

**DOI:** 10.3389/fmed.2024.1421109

**Published:** 2024-10-03

**Authors:** Yiqi Huang, Tianxiao Fu, Yanling Zhang, Weigang Shen, Weiwei Sang, Meixiang Han, Fang Wang, Fenjuan Chen

**Affiliations:** ^1^Department of Nephrology, Shaoxing Second Hospital, Shaoxing, Zhejiang, China; ^2^Department of Traditional Chinese Medicine, The First Affiliated Hospital of Zhejiang University, Hangzhou, Zhejiang, China; ^3^Department of Dermatology, Shaoxing Second Hospital, Shaoxing, Zhejiang, China

**Keywords:** icodextrin, severe allergic rash, acute localized exanthematous pustulosis, peritoneal dialysis, peritoneal dialysate

## Abstract

**Background:**

Icodextrin is a type of peritoneal dialysis (PD) osmolyte that can be extended retention times (8–16 h) and may offer a viable alternative to conventional glucose dialysis solutions for PD patients. Nonetheless, prolonged use of icodextrin may lead to allergic rash, and rarely severe skin lesions.

**Case presentation:**

In February 2024, a 45-year-old male was admitted to the Department of Nephrology at Shaoxing Second Hospital presenting with a 3-day history of intense generalized pruritic erythematous rash. Physical examination revealed diffuse erythematous pruritic rash and exfoliative rash, particularly on the abdominal. Abnormal laboratory findings included elevated eosinophil count and total IgE levels, indicative of an allergic rash. Standard anti-allergic regim was initiated. However, on the third day in the hospital, the patient developed new pustules on his neck and arms. Subsequent historical investigation uncovered that the individual had previously administered icodextrin 2 weeks prior due to volume overload, and the last intraperitoneal administration time was second day of hospitalization. The dermatologist rendered a diagnosis of generalized exfoliative rash and acute localized exanthematous pustulosis (ALEP) induced by icodextrin, and initiated prophylactic antimicrobial therapy accordingly. Furthermore, the patient declined to undergo a skin biopsy. Noteworthy is the observation that the rash ameliorated and the pustules resolved by the seventh day post-admission. Presently, the patient is still under clinical follow-up.

**Conclusion:**

This article aims to report the first case of severe allergic rash caused by icodextrin in Chinese PD patients and highlight the potential for icodextrin to trigger ALEP. A literature review of similar cases found that severe allergic rash induced by icodextrin is rare, the underlying mechanism remains poorly understood, and the prognosis is positive with proper treatment.

## 1 Introduction

Icodextrin, the water-soluble glucose polymer derived from starch and linked by *α*-1 and α-4 glycosidic bonds, exhibits isotonic properties, low glucose content, and minimal metabolite presence (1). Since its introduction to the European market in 1997, icodextrin peritoneal dialysis (PD) solution has been extensively utilized in over 80 countries globally and has demonstrated favorable clinical efficacy and safety (2, 3). In developed nations, the utilization rate of icodextrin among PD patients exceeds 50% (4). Nonetheless, prolonged use of icodextrin may lead to allergic rash, and rarely severe skin lesions. The risk of rash induced by icodextrin is reported to be approximately 2–3 times higher than that associated with glucose-based dialysate (5). This article outlines the first documented case of a severe skin allergy in China following the use of icodextrin, leading to generalized exfoliative rash and acute localized exanthematous pustular (ALEP).

## 2 Case presentation

**Figure 1:**
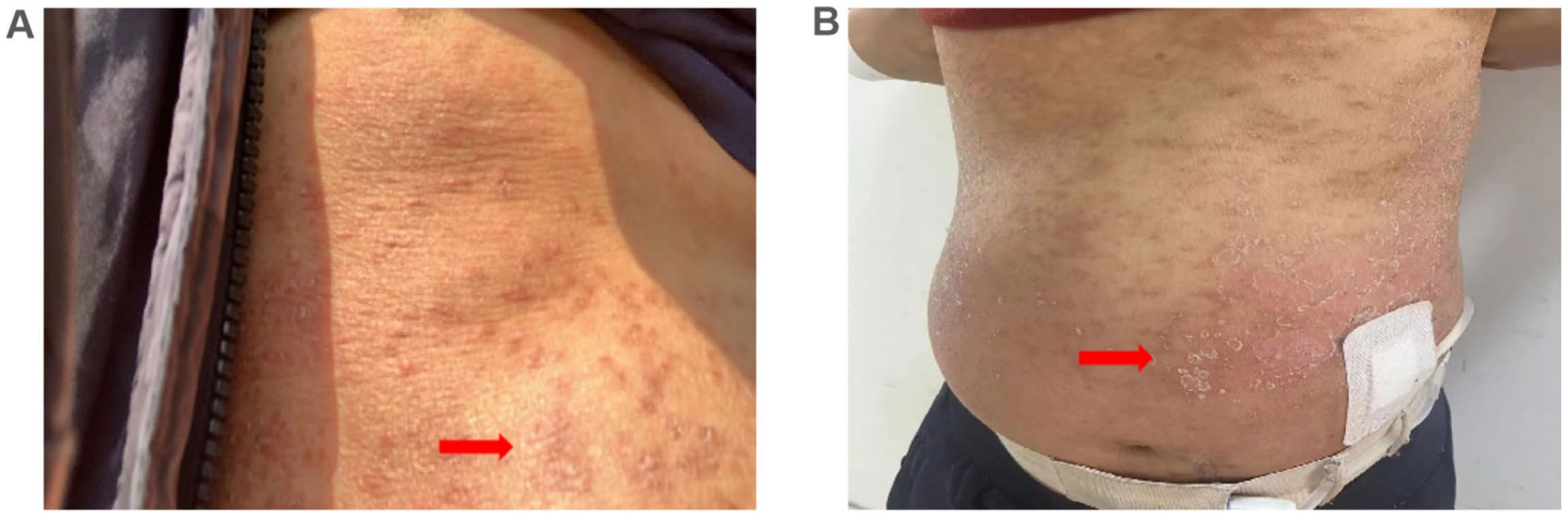
Skin manifestations. **(A)** Pruritic erythematous rash developed on the abdomen 3 days prior to admission (arrows); **(B)** Generalized pruritic erythematous rash and exfoliative rash developed on admission (arrows).

On February 2, 2024, a 45-year-old male undergoing PD was admitted to the Nephrology Department of Shaoxing Second Hospital with a 3-day history of intense generalized pruritic erythematous rash (Figure 1A). Routine physical examination on admission revealed blood pressure of 141/85 mmHg, body temperature of 37.3°C, diffuse erythematous pruritic rash, and exfoliative rash, particularly on the abdominal (Figure 1B), without oral and mucosal lesions.

The patient’s daily oral medication regimen consisted of sacubitril/valsartan sodium (200 mg, once daily), nifedipine controlled-release tablet (60 mg, once daily), roxadustat (120 mg, three times a week), and Calcicort D tablet (600 mg, once daily). He consistently underwent a standard continuous ambulatory peritoneal dialysis (CAPD) protocol, which involved three exchanges of 2,000 mL of 1.5% PD solution and one exchange of 2,000 mL of 2.5% PD solution per day. No changes were made to the treatment regimen from the initial PD to the onset of the rash. Additionally, neither recent travels nor allergies were noted in the patient’s medical history.

Laboratory tests include elevated non-specific allergy indicators (eosinophil count 0.75 × 109/L, IgE 315 IU/mL), decreased nutritional markers (hemoglobin 9.9 g/dL, albumin 31 g/L), disordered electrolytes (serum potassium 3.7 mmol/L, serum sodium 135 mmol/L, serum calcium 1.96 mmol/L, and serum phosphorus 1.34 mmol/L) and normal inflammatory markers [white blood cell (WBC) count of 8.68 × 109 cells/L, C-reactive protein (CRP) 3.6 mg/L, procalcitonin (PCT) 0.01 ng/mL, WBC count in peritoneal dialysate of 0/mm3]. CT scan of the chest and abdomen showed no obvious abnormalities.

**Figure 2:**
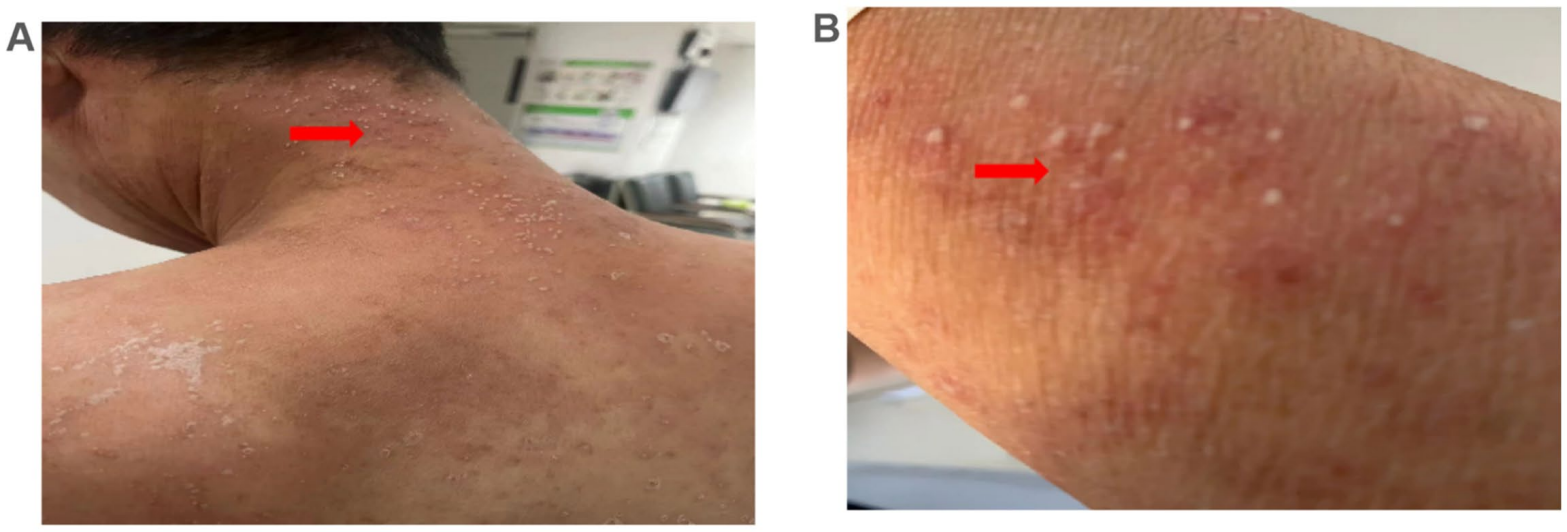
Microbiological examination results. **(A,B)** Multiple non-follicular pustules on an erythematous base were observed on the neck and arm 3 day after admission (arrows).

**Figure 3:**
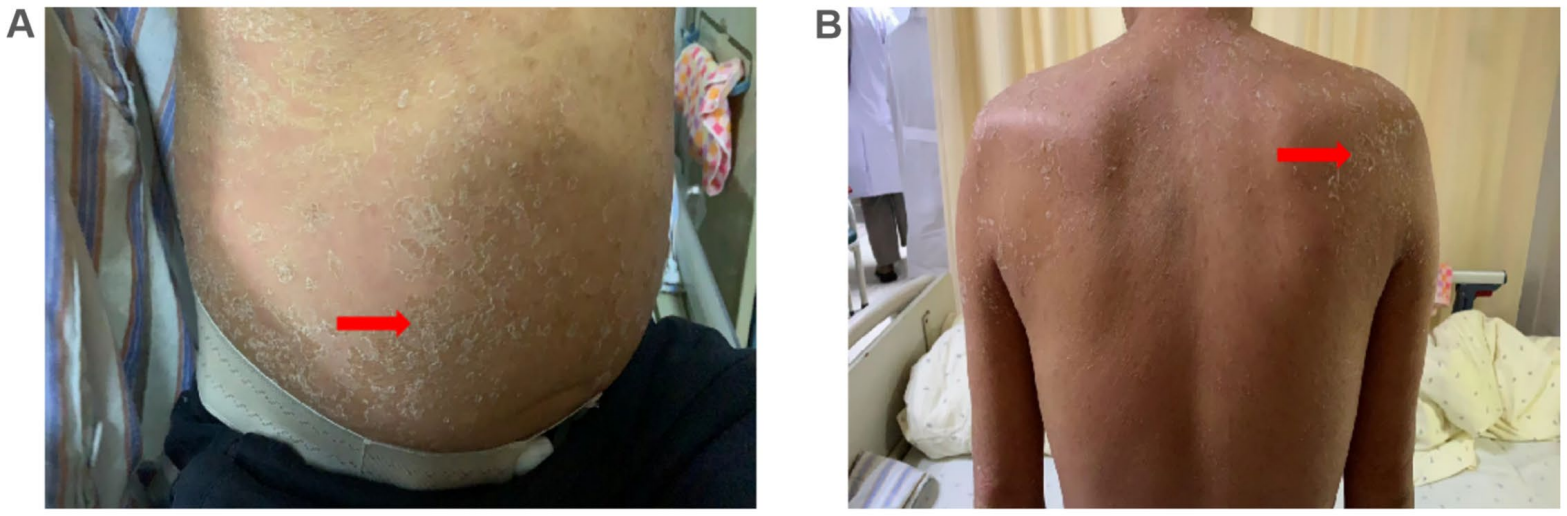
Skin manifestations. **(A,B)** Exfoliative rash on the abdomen and neck improved 7 days after admission (arrows).

**Figure 4:**
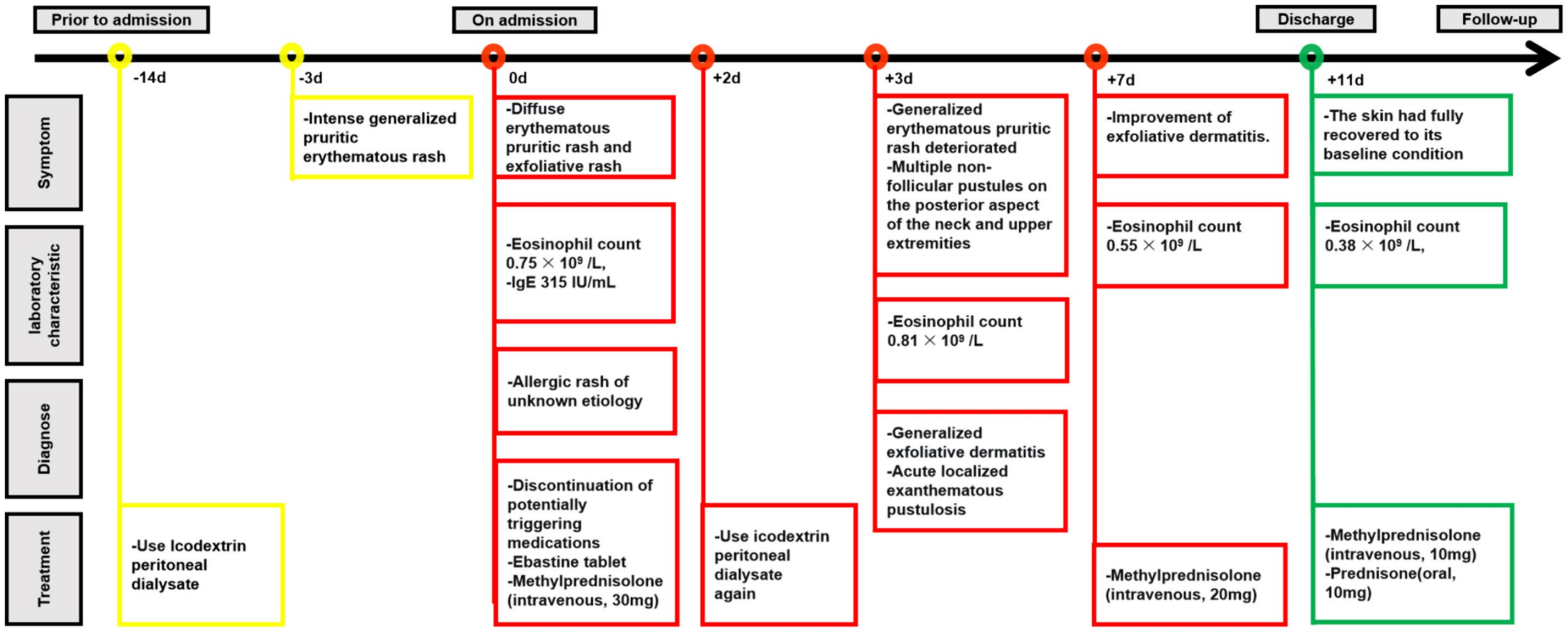
The timeline for diagnosis and treatment.

The patient was hospitalized with an initial diagnosis of allergic rash of unknown etiology and was treated with a standard anti-allergic regimen, consisting of discontinuation of potentially triggering medications (excluding antihypertensive medications and dialysis fluids), administration of ebastine tablet (oral, 10 mg/dose, once daily), and methylprednisolone (intravenous, 30 mg/dose, once daily). On the third day post-admission, the patient’s generalized erythematous pruritic rash deteriorated, with the emergence of multiple non-follicular pustules on the posterior aspect of the neck and upper extremities (Figures 2A,B). Upon further investigation, he initiated the use of icodextrin on an alternate day schedule 2 weeks prior due to volume overload and the last intraperitoneal administration time was second day of hospitalization. The dermatologist, who took into account patient’s allergic history and the manifestation of skin rash, rendered a diagnosis of icodextrin-induced generalized exfoliative dermatitis and ALEP. As a prophylactic measure against infection, mupirocin ointment (external application, once daily) and piperacillin-tazobactam (intravenous, 4.5 g/dose, twice a day) were incorporated into the therapeutic regimen. Despite a suggestion for a skin biopsy, the patient opted not to proceed any invasive procedures. By the seventh day following admission, the patient exhibited improvement in exfoliative rashes, accompanied by a reduction in pustules (Figures 3A,B). By the 11th day post admission, the patient’s skin had fully recovered to its baseline condition. Upon discharge, this patient remained free from allergic rash and continues to be under clinical follow-up. Figure 4 illustrates the timeline for diagnosis and treatment.

## 3 Discussion and conclusion

Icodextrin, a polymer derived from starch composed of differing glucose chain lengths, functions as an osmotic agent capable of substituting conventional glucose dialysis solutions and has benefits that encompass heightened ultrafiltration, maintenance of peritoneal integrity and functionality, enhanced biocompatibility, and improved survival outcomes (6, 7). In addition, the utilization of icodextrin in individuals undergoing PD with concurrent diabetes or refractory congestive heart failure has demonstrated efficacy in the management of blood glucose levels and improvement of cardiac performance (8–10).

While the efficacy and safety of icodextrin are well established, adverse reactions like peritonitis and allergic rash can still occur during its use. The incidence of allergic rash from icodextrin ranges from 2.3 to 18.9%, with severe cases being rare (6, 11, 12). A multicenter, randomized, double-blind study involving 92 PD patients found a significantly higher rate of maculopapular eruptions in the icodextrin group (4.6%) compared to the glucose dialysate group (0%) (5). However, a 2013 meta-analysis that included 11 randomized controlled trials with 1,222 participants did not find a statistically significant increase in eruption risk associated with icodextrin vs. glucose dialysate (6). Thus, the debate over whether long-term use of icodextrin raises the risk of allergic rash continues. The precise pathophysiological process by which allergic rash induced by icodextrin is not yet fully understood. One proposed mechanism suggests that icodextrin is metabolized within patients, leading to the formation of maltose molecules with a glucan-like configuration, and maltose molecules may accumulate in the skin and peripheral nerves, combining with immunoglobulin G molecules to form immune complexes, ultimately provoking sustained allergic responses, which is analogous to the anaphylactic response elicited by glucan (13, 14).

To the best of our knowledge, a total of 11 cases documenting severe allergic rash induced by icodextrin have been comprehensively reported in the existing literature. These cases include three from France (15), two from the United States (16, 17), and one each from Saudi Arabia (18), Greece (14), Turkey (19), Canada (20), South Korea (21), and the United Kingdom (22). This case is the first reported icodextrin-induced severe allergic rash in China, which may be related to the time of icodextrin’s market approval. Icodextrin was only officially approved for the Chinese market in August 2021, while it has been used in Europe and the United States for more than 20 years. Table 1 provides a comprehensive summary of 11 cases. Icodextrin-induced severe allergic rash primarily impacted female patients (8/11), ranging in age from 23 to 91 years. Research has indicated that female is a significant risk factor for allergic rash resulting from icodextrin exposure, however, there is no observed correlation between gender and prognosis (23). The time interval between the use of icodextrin and the onset of severe rash in almost all cases (10/11), including our case, varied from a few days to 4 weeks. Only one PD patient documented an immediate allergic response following exposure to icodextrin, as detailed by Lee (21), which is exceptionally uncommon. The rash types were primarily categorized as generalized exfoliative rash (7/11) (14–18, 21, 24) and purulent erythematous rash (3/11) (14–18, 21, 24), with only the patient described by Valance et al. being diagnosed with Acute Generalized Exanthematous Pustulosis (AGEP) (15). The rash type in our case is rare, presenting as a generalized exfoliative rash and ALEP, with no previous reports of ALEP induceded by icodextrin. ALEP is a unique form of Acute Generalized Exanthematous Pustulosis (AGEP), marked by nonfollicular, pinhead-sized pustules in localized skin areas (25). Research indicates that about 90% of ALEP cases are due to systemic drug use, often affecting the face, neck, and other regions (26). This ailment typically resolves spontaneously with prompt cessation of the medication (25). Nevertheless, the exact pathological mechanism of ALEP is not fully understood and may bear resemblance to ADEP, primarily characterized by T cell-mediated drug-specific mechanisms that trigger delayed-type allergic responses. In our case, ALEP presented on the third day of hospitalization; icodextrin was promptly ceased and corticosteroid therapy was commenced, leading to a positive clinical outcome. Regrettably, a positive patch test could not be conducted due to the patient’s acute skin lesions and continued anti-allergic therapy. Additionally, the overall prognosis of icodextrin-induced rash was favorable, with the exception of specific cases such as the patient described by Alotaibi who necessitated transfer to hemodialysis due to refractory peritonitis (16), and the elderly patient reported by Liakopoulos who tragically passed away as a result of an accident (colon rupture) during treatment for the rash (14).

**Table 1:** Case summary of icodextrin-induced severe allergic rash.

References	Country	Gender	Age	Duration of icodextrin	Distribution of rash	Duration of rash	Diagnose	Outcome
Alotaibi et al. (16)	United States	Female	77	2 weeks	Chest, arms, abdomen, and back	1 month	Generalized exfoliative rash	Switch to hemodialysis
Khatib et al. (18)	Saudi Arabia	Male	43	1 day	Whole body	4 weeks	Generalized exfoliative rash	Continued PD
Liakopoulos et al. (14)	Greece	Female	91	15 days	Whole body	7 days	Generalized exfoliative rash	Died of colonic rupture
Cevher et al. (19)	Turkey	Female	23	1 day	Neck and upper extremities	1 week	Pruritic erythematous rash	Continued PD
Almiani et al. (17)	United States	Male	56	2 weeks	Torso and limbs	Within days	Generalized exfoliative rash	Continued PD
Valance et al. (15) case 1	France	Female	50	11 days	Palms and soles	1 week	Pruritic erythematous rash	Continued PD
Valance et al. (15) case 2	France	Female	45	13 days	Whole body	15 days	Acute generalized exanthematous pustulosis	Continued PD
Valance et al. (15) case 3	France	Male	45	12 days	Palms and soles	Unknown	Exfoliative rash	Continued PD
Ankur et al. (20)	Canada	Female	50	4 weeks	Trunk and back	2 weeks	Pruritic erythematous rash	Continued PD
Lee et al. (21)	Korea	Female	85	Immediately	The whole body	7 days	Generalized pruritic erythematous rash	Continued PD
Fletcher et al. (22)	United Kingdom	Female	61	14 days	Chest, trunk, arms, and legs	14 days	Generalized pruritic erythematous rash	Continued PD
Present report (2024)	China	Male	45	2 weeks	Whole body	11 days	Generalized exfoliative rash and ALEP	Continued PD

In summary, icodextrin has been safe and well-tolerated in Chinese PD patients for the past 3 years. However, rare complications like severe allergic rash require attention. This report documents the first case of icodextrin-induced severe allergic rash in China, identifying icodextrin as the cause of ALEP. While no standardized guidelines exist for diagnosing and treating ALEP, early diagnosis and prompt treatment usually result in positive outcomes for PD patients.

## Data Availability

The original contributions presented in the study are included in the article/supplementary material, further inquiries can be directed to the corresponding author.
